# Socio-conversational systems: Three challenges at the crossroads of fields

**DOI:** 10.3389/frobt.2022.937825

**Published:** 2022-12-15

**Authors:** Chloé Clavel, Matthieu Labeau, Justine Cassell

**Affiliations:** ^1^ LTCI, Telecom-Paris, Institut Polytechnique de Paris, Paris, France; ^2^ School of Computer Science, Carnegie Mellon University, Pittsburgh, PA, United States; ^3^ Inria, Paris, France

**Keywords:** Socio-conversational systems, Natural language processing, Machine learning, Multimodality, Affective computing, Social signal processing

## Abstract

Socio-conversational systems are dialogue systems, including what are sometimes referred to as chatbots, vocal assistants, social robots, and embodied conversational agents, that are capable of interacting with humans in a way that treats both the specifically social nature of the interaction and the content of a task. The aim of this paper is twofold: 1) to uncover some places where the compartmentalized nature of research conducted around socio-conversational systems creates problems for the field as a whole, and 2) to propose a way to overcome this compartmentalization and thus strengthen the capabilities of socio-conversational systems by defining common challenges. Specifically, we examine research carried out by the signal processing, natural language processing and dialogue, machine/deep learning, social/affective computing and social sciences communities. We focus on three major challenges for the development of effective socio-conversational systems, and describe ways to tackle them.

## 1 Introduction

A number of different communities have taken on the task of developing conversational systems: primarily researchers in human-agent interaction, machine learning, natural language processing (NLP), dialogue, and in affective/social computing. Unfortunately, like the story of the blind men and the elephant^1^, each of these communities has come at the topic from a different angle, and has therefore had difficulty seeing the larger picture. As a simple example, in order to understand emotion, the NLP and affective computing communities have come from different places. The affective community initially focused on non verbal expressions (e.g., facial, head) of emotions while the NLP community initially developed the notion of sentiment analysis without reference to nonverbal behavior. As a demonstration, only one paper about verbal behavior is found out of the whole proceedings of the first conference in Affective Computing (Tao et al., 2005), while the domain of sentiment analysis appeared in 2002 (Turney, 2002) started to include non verbal aspects 10 years later with the apparition of “multimodal sentiment analysis” (Morency et al., 2011). Because of these different histories, as both communities begin to focus today on multimodal behavior, their approaches are sometimes limited by the means they have developed to address only one modality. Another example comes from research carried out in either natural language recognition or generation, while more recent research is trying to jointly handle recognition and generation with end-to-end systems (Serban et al., 2016). In addition, over the course of history the different fields have been differentially affected by social science research (including pragmatics and psychology), which provides keys for understanding conversational phenomena.

**FIGURE 1:**
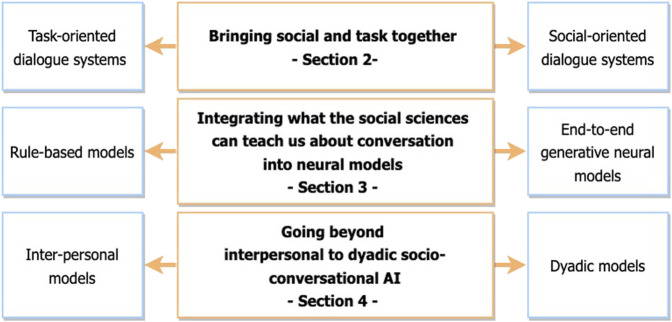
The three challenges for gathering different research in conversational AI.

Happily, there is increasing awareness of the need for better communication among research domains, and for the fields to cohere around a unified approach to socio-conversational systems. In recognition of this fact, major machine learning and NLP conferences such as AAAI, IJCAI, and ACL now have frequent satellite workshops on Affective and Social computing (e.g., AAAI-20 Workshop on Affective Content Analysis, ACL-2020 SocialNLP Workshop, IJCAI-2021 Workshop on Competitive and Cooperative Social Interactions, NAACL-2022 Bridging Human–Computer Interaction and Natural Language Processing Workshop). Nevertheless a schism remains, and we believe that the field will be more productive once a series of challenges shared across the research domains are identified. As summarized in Figure 1, this paper aims to point out three major challenges, and describes ways to tackle them.

The first challenge (Section 2) aims to better link research on task-oriented systems with research on social-oriented systems. There is increased agreement, as we described here, that we must bring social and task goals together for more effective conversational systems, as the social dimension is always present in a human-agent interaction, as in human-human conversation, and chat with someone not indicating that they are engaged gets boring after a short while. We need new architectures that better interleave task and social and that leverage research from both sides.

Because socio-conversational systems need to provide socially-relevant answers for a given task, as argued within the first challenge, methods underlying their development must allow an easier and controlled integration of social science knowledge. Two very different types of approaches co-exist for the integration of both task and social components into socio-conversational systems: approaches attracted to end-to-end deep learning models (bottom-up) and approaches based on reasoning and derived from knowledge-based systems (top-down). In Section 3, we argue, along with others in the field, that we thus need to tackle a second challenge: the development of new hybrid architectures that leverage research from both rule-based and machine learning (including neural models). How Symbolic AI and deep learning approaches can co-exist is an issue addressed in other areas than socio-conversational systems. It lies at the core of all the research carried out in Artificial Intelligence. We approach it here in the context of research on socio-conversational systems, as they raise particularly interesting issues due to the nature of the knowledge that needs to be integrated into the neural models.

Finally, we argue that the social functionality of socio-conversational systems should be revisited. We must go beyond computational studies that look at both interaction participants as separate individuals trading meanings (what we refer to as an interpersonal approach). We must leverage what social science research tells us about the dyadic nature of social phenomena. This leads us to the third challenge: socio-conversational systems must employ neural architectures that mirror these dyadic processes by analyzing the user’s states and generating the agent’s utterances within the dyadic process of two interlocutors working together^2^. We believe that, in tackling these three challenges, and in tackling them together, socio-conversational systems will indeed evolve to be true partners to their human users.

## 2 Challenge 1: Bringing social and task together

The recent scientific literature in conversational systems is often clearly divided according to the end purpose of the system, which is generally one of two possibilities: task-oriented dialogue, where the system helps the user to accomplish a specific task, or more general purpose or open domain or chat dialogue systems (Huang et al., 2020) where the goal is simply to engage the user. For both, tools from deep learning, made possible by the growing availability of conversational data, have fostered a significant body of work. While both types of systems can be similarly described as finding the best answer to a user’s utterance, they do not address the same challenges nor do they usually follow the same conversational structure. In what follows we first describe the general lines of work on task-oriented systems, and then turn to social-oriented systems, before finally discussing why we should bring social and task together and what it would take to do so.

### 2.1 Task-oriented systems

The purpose of the very first dialogue systems was to use their knowledge to complete a given task for the user. A typical example of these early systems is Trains, where users could ask for help in booking train travel and the system, using a slot-filling approach, could recommend specific trains (Ferguson, 1996).

The concept of task-oriented systems encompasses a wider variety of tasks that can be viewed as question answering problems in different application domains (e.g., a train travel reservation, customer relationship management, movie recommendations). These can be either text-based, spoken, or multimodal. The relevant dialogue responses are generated or selected according to the task, and these first task-oriented systems ignored questions of social suitability, for example smiling in the face of a user’s frustration.

**FIGURE 2:**
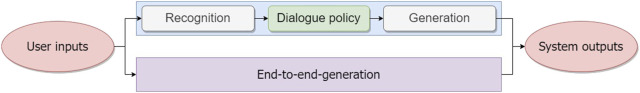
Two types of task-oriented systems architectures: modular (top) or end-to-end (below).

In the area of task-oriented systems, some researchers work on generation, some on recognition, some on systems that include both recognition and generation modules, and some include both in a single process. Figure 2 shows the two types of architectures currently used in task-oriented systems. The first type is modular and includes three main modules (Mehri et al., 2019): i) the dialogue understanding or recognition^3^ part assigning the user input to labels that are sometimes dependent (such as book a train) and sometimes independent of domain (such as ask a question); ii) the dialogue policy, selecting the system’s type of answer (for example a suggestion of a particular train) given the previous dialogue understanding output; and, iii) generating or selecting the text corresponding to the selected dialogue policy. The generation of natural language in these modular systems is accomplished with templates or language models. And underlying the dialogue policy module is often a planning structure that establishes how to move from one task to another in order to achieve the user’s goals (Rich and Sidner, 1997).

The second type of task-oriented systems is the neural conversation models trained end-to-end (Ham et al., 2020), aiming to directly select or generate the relevant system answer according to user inputs. The generation of the answer is done *via* a language model, which is given the representation of the question as input. These architectures are primarily used for open-domain question answering [based for example on logical and common-sense reasoning (Helwe et al., 2021)]. Their applicability to specific domains (e.g., train reservations) is limited by the amount of data available, even though recent research allows mitigating this issue using pretrained language models and few-shot learning (Wu et al., 2020).

### 2.2 Social-oriented systems

From Weizenbaum’s Eliza system onwards (Weizenbaum, 1966), systems have been developed where the first goal is to engage a user when answering open-domain questions such as asking the weather. Perhaps the most well-known of these systems today are Microsoft’s Tay [or, in Chinese, XiaoIce (Zhou L. et al., 2020)], ICONIQ’s Kuki^4^, and Google’s LaMDA (Thoppilan et al., 2022). Regardless of whether the systems use text, spoken voice, or a voice plus a body, the criterion of success here is to keep the user engaged for as long as possible.

Similarly to task-oriented systems, some social-oriented systems have concentrated on recognizing and understanding these phenomena, while others have concentrated on their generation (Cassell, 1998). Those focusing on either recognition or generation, may simply integrate the visible/audible behaviors, such as emotion words, prosody, facial expressions, hand gestures and eye gaze shifts (Schuller et al., 2002; Clavel et al., 2008; Zadeh et al., 2017). Or they work on a social dialogue policy module which consists rather in defining suitable socio-emotional strategies or mechanisms such as empathy (Ma et al., 2020) and incorporating information about the user’s emotional state and resulting behaviors. In this latter line of research, we can cite Bui et al. (2010) and the empathetic social chatbot XiaoIce (Zhou L. et al., 2020) that uses both Markov Decision Processes (MDP), or Pecune and Marsella (2020) and Ritschel et al. (2017) using a social reward in reinforcement learning. Complete modular architectures fall into this latter category, integrating underlying cognitive or socio-cognitive structures (such as emotion, or sentiment) and then instantiating them in surface-level observable behaviors, such as Greta (Niewiadomski et al., 2009), Fatima (Dias et al., 2014), or SARA (Matsuyama et al., 2016).

More recently, researchers working on end-to-end approaches (see Figure 2) have also started to work on social-oriented systems - although, for the moment, these systems exist only in text (Zhong et al., 2020; Liu et al., 2021). They dispense with the steps of classifying the user’s utterance and identifying the relevant dialogue policy. Hence, they can be used indifferently for non-task specific systems, or task-oriented systems, if corpora of conversations in the relevant domain are available. This requires the models to be able to implicitly integrate the status of the social relationship between the human and the agent in an interaction without the need for supervision either to measure the user’s state or to generate a socially-relevant answer.

As we will see in more detail in Section 3.2, one approach is to explicitly model the status of the social relationship in end-to-end generation models such as DialogGPT (Liu et al., 2021). For example, Zhong et al. (2020) learn a neural model (Bert model named CoBERT) on the basis of empathetic conversations for response selection. Liu et al. (2021) use DialoGPT with supervision to generate the agent’s response as a condition of conversational strategies. In Section 3, we will also discuss the advantage and drawbacks of such approaches in socio-conversational systems.

### 2.3 Why should we bring social and task together and what would it take to do so ?

Numerous studies have shown the impact of social bonds on task performance (Dörnyei and Kormos, 2000; Kamdar and Van Dyne, 2007). Children learn better when paired with friends, doctors enroll more study participants when they create a bond with their patients and, of course, salespeople sell more when their customers feel close to the person doing the selling. Not only are these bonds impactful, they are also ubiquitous. In human-human interaction, the social dimension plays a striking role, regardless of the location, the interlocutors, or the nature of the work that the interlocutors might be attending to. In fact, some have argued that language itself was developed in order to serve the purpose of creating social bonds (Dunbar, 1998). If dialogue systems are to spend any time with people, we might think that they should also be able to fluidly move from task to social and back again.

This does not mean that every conversational system should bare its (robotic) soul, and ask about the customer’s private life. A system implemented at the World Economic Forum showed, unsurprisingly, that some world leaders simply wanted information from the system, and not to engage in social interaction (Pecune et al., 2018). However, it is difficult to think of a case in which a system should not be capable of listening and responding to frustration and other psychological states. Nevertheless, as we have noted above, systems have long been divided into those with a task focus and those with a social focus and attempts to make task-focused systems more social often ignored the existing literature on social-focused systems.

To integrate a social component to task-oriented systems effectively, indeed, is going to entail an understanding of how social phenomena operate: the multimodal forms that are associated with them, and the social functions that underlie them. We argue that in order for this to happen, research on task-oriented systems may need to be more attentive to research in the social sciences on the many-to-many mappings between social phenomena such as rapport, and the multimodal forms that carry them, such as teasing, embarrassed laughter, and smiles.

Those with a social focus sometimes integrate a task as well in order to demonstrate that the integration of the social aspects allows increased efficiency on a task. In this way, Bickmore et al. (2013) and then Lee and Choi (2017) integrate conversational behaviors dedicated to strengthening social bounds with the user with the view to improving effectiveness for a museum guide and movie recommendation agent, respectively. Even in these cases, which could be considered mixing task and social, the task and the social components are treated independently and the tasks considered are always rather “socially oriented”.

Thus, what we need is a new kind of system with architectures that better interleave task and social functionality, and the modalities that carry them. As modular architectures are used in both types of systems, the integration of social and task can be done in a relatively simple way. For example, at the level of the policy dialogue module, reinforcement learning methods can optimize the response according to a reward that incorporates both a social and a task-related goal (Pecune and Marsella, 2020). As far as end-to-end architectures are concerned, their underlying paradigm is to implicitly integrate task and social together. But the implicit modeling of the social component as modeled in data whose content we do not control is hazardous for socio-conversational systems in production. How do we ensure that our system adopts a strategy that is socially relevant, timely (we don’t want the system’s response to be always empathetic), and that will serve the task, when using end-to-end generation models? First, we need to identify the conversational strategies that serve the task (e.g., emotional support for customer service agents). Second, we need to generate an answer relevant to the task and communicated with the appropriate conversational strategy. We saw in Section 2.1 that end-to-end models can provide answers relevant to the task and in Section 2.2 that some methods exist for conditioning the answer to the relevant conversational strategy. Integrating task and social relevance together, could thus be done by reranking task-relevant answers according to their socially-relevance or by conditioning the generation process jointly to the task and the conversational strategy.

Interestingly, recent research focuses on the safety of the answer in order to avoid inappropriate answers and social norm violations by using annotated data and external knowledge sources (Thoppilan et al., 2022). This raises important ethical questions, as the definition and coding of what constitutes the social norm is not only task-dependent but also culture-dependent and may rightly be controversial (Ferrari et al., 2016).

Lastly, this brings us to the broader issue of the evaluation of conversational systems. Current research defines two ways of evaluating conversational systems at the conversation level: 1) qualitative evaluations based on a user’s or an external observer’s perception of the conversation according to different criteria that are either task-oriented (such as resolving a user’s problem for customer relationship conversational systems) or socially-oriented [such as a user’s self-reported engagement (Sidner et al., 2005; Clavel et al., 2016)]; 2) automatic measures of the task efficiency (such as the learning gain for educational applications (Norman et al., 2022) or the satisfaction gain or loss for customer relationship applications). Whether the measures are qualitative or automatic, to evaluate conversational systems bringing social and task together, we need to find a way to better interact task-related criteria with socially-oriented criteria. But, depending on the purpose of the conversational system, we have to wonder whether to focus on the evaluation of the task saying that the social is here to serve the task, or evaluate the two aspects both independently and jointly.

## 3 Challenge 2: Mixing neural and social-science derived models

In the previous section, we saw that the social component is crucial for most conversational systems that will engage for longer than a few minutes, and that, to integrate it, we need to integrate knowledge of social phenomena. The challenge that we propose to tackle here concerns the approaches underlying the taking into account these social phenomena in conversational systems. In this regard, the different histories of the research domains investigating socio-conversational systems create a tension between research based on rules, that may be more conducive to the integration of knowledge from the social sciences (presented in Section 3.1) and research relying on end-to-end deep learning approaches (presented in Section 3.2). Between these two extremes is a continuum depending on how much the social science knowledge is implicated in the design of the socio-conversational systems, from an explicit modeling of the knowledge to fully data-driven machine learning approaches. We examine this continuum and the possible integration of the two perspectives in Section 3.3.

### 3.1 Rule-based models

Explicit rule-based models are often derived from theories of human communication. In fact, these systems are often instantiations of social science theories, although not necessarily exact or accurate ones. Such approaches do not require any data on the front end, although a proper evaluation would require data on its use by people.

In this line of research, computational linguists working on rule-based analysis of user’s utterance rely on linguistics. They define rules capturing regularities that, on the basis of strings of words, reveal correlations between linguistic categories and meanings (Neviarouskaya et al., 2007; Taboada et al., 2011; Langlet and Clavel, 2015). They rely on research in linguistics on the content of language - its semantics and syntax, and how they are deployed in expressing stances and sentiments (Martin and White, 2003). For example, a speaker might use indirectness to express the stance of lack of confidence in the truth value of a statement, or use negation, intensifiers, conditional tense, or metaphors to express sentiments such as negativity.

The methods underlying the implementation of dialogue policies and response generation have also historically been rule-based. Basic rules are used in Bickmore et al. (2013) in order to implement an empathetic agent. The rules implicated verbal and non verbal behaviors of both user and agent. Skowron et al. (2011) use AIML (Artificial Intelligence Markup Language) for defining response templates for agent’s utterance according to the user emotions. Linguistic templates and hierarchical task networks are used in Campano et al. (2015) in order to implement alignment strategies. Systems exist that took Duncan’s rules (Duncan, 1972) on how speaker turns are managed between people, using eye gaze shifts, and translating them into rules that decree that the speaker should look at the listener when offering the turn (Cassell et al., 1999).

Some rule-based models rely on hand-annotated data, where the annotation schemes are derived from pre-existing theories or bottom-up perspectives on conversation [such as grounded theory (Glaser et al., 1968)]. After annotation is completed, probabilistic rules are derived by hand from statistical analysis. These models are in themselves forms of social science theory, and may be published as such, as they provide an explanation for the appearance of particular behaviors in conversation. They may be also published as the basis for computer science algorithms where they describe the right behavior for a system to undertake on the basis of a user’s conversational moves. They require a corpus large enough to allow good statistical power, but nowhere near as large as is required for non feature-based models. For example, analysis of a corpus of conversations revealed that Duncan’s eye gaze rules were insufficient as they only took turn-taking into account (Cassell et al., 1994). Thus when new information is introduced by speakers, eye gaze shifts towards the listener X% of the time. The end of a turn also evokes an eye shift at Y% of the time, and when the end of the turn and the introduction of new information co-occur within two words of one another, eye gaze shifts towards the listener 100% of the time.

Developing rules is time consuming and may be too corpus- or domain-specific but rules have the potential to provide an explicit formalization of social science research and are efficient when little data is available.

### 3.2 Machine learning models

**FIGURE 3:**
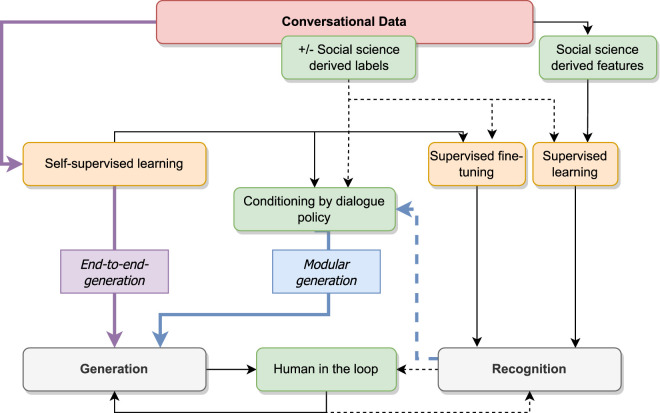
Machine learning approaches in socio-conversational systems. Orange boxes represent the different types of supervision of machine learning models, white boxes the different usages, green boxes the intervention of external knowledge, and blue and purple arrows represent the modular and end-to-end settings, respectively. Dotted arrows indicate when the information comes from labels derived from human knowledge.

In recent years, rule-based approaches have been gradually supplanted by machine learning approaches. The question that arises is their capacity to integrate knowledge from the social sciences. To answer this question, Figure 3 examines how such knowledge is integrated into the different machine/deep learning models involved in socio-conversational systems by using dotted arrows. As seen in Section 2, the generation can rely on previous recognition of users’ utterances and dialogue policy modules (in modular approaches) or not (end-to-end generation). The figure indicates the two usages of machine learning models: analysis/recognition of a user’s utterance or generation of an agent’s utterance.

First of all, let’s look at the most recent systems that are completely free from external knowledge and supervision–namely the end-to-end generation models represented by purple arrows in Figure 3. Serban et al. (2016) proposed one of the first end-to-end neural system dedicated to dialog, based on recurrent neural networks. Since then, end-to-end generative neural models have in the last few years been used more and more for end-to-end generation in dialogue systems. Such models are inspired by the use of neural networks for generative language models, and applications like machine translation. They jointly learn to represent input data, thus only needing unlabeled conversations to be trained. Hence, they do not suffer from the same data scarcity, and can be used indifferently for social-oriented or task-oriented systems, if corpora with conversations of the relevant domain are available, such as done in DialoGPT (Zhang et al., 2019). The power of such models is that they don’t require external knowledge or social science-derived features to be built. However, the dialogue policy underlying the response is not known and could not be controlled. This requires the models to be able to implicitly integrate the status of the social relationship between the human and the agent in an interaction.

In modular approaches (retrieved by the blue arrows in Figure 3), the social science knowledge is intervening at two different stages: in the supervision of machine learning models or in the design of social-science derived features. Supervised machine learning models are learned on the basis of an annotated corpus (here seen as raw data with labels corresponding to what the system wants to detect or generate). For example, a chat conversation might be annotated for the emotions that the participants are putatively feeling, as decided by crowdworkers, at the level of each turn. Here notions from the social sciences find their way into the process by means of the labels that are attributed to each turn (such as happiness, sadness, frustration, etc.), and that the machine learning algorithm must use as supervision of its learning process, along with the raw chat data (Clavel and Callejas, 2015). The task of providing a relevant set of labels, however, is not straightforward. The description used for the supervision needs to be sufficiently rich to reveal the complexity of the putative underlying speaker states (emotions, in the case of our example), as perceived by the crowdworking annotator. At the same time, supervised machine learning requires a certain simplicity in the labels - there must not be too many of them, nor can there be overlap amongst them, and each label must be attributed to roughly equal amounts of the data. In the case of generation, the labels may be used to condition the generation of the text, voice, gestures or facial expressions of the agent. A set of labels, such as emotion categories or social stances are initially chosen and the dialogue policy consists of choosing the label among this set that will be used for the generation.

In terms of the features (such as eye gaze, prosody, or groups of words, for example) that the model will associate to labels, these can be annotated by hand, automatically extracted, or come from a prior machine learning process that creates a representation on the basis of unannotated data. The choice of hand-annotated or automatically extracted features has more to do with how easy it will be to transform this hand-annotation process into something autonomous. However, in both the case of hand-annotated and software-extracted, the features depend on pre-existing theory about what is important (social-science derived features). For example, one might hand-annotate eye gaze shifts, or extract them using software such as OpenFace (Baltrušaitis et al., 2016). Both Openface, and the manual given to annotators come from reading the social science literature on eye gaze. Regarding audio modalities, pitch is a feature that is largely used in emotion recognition systems (Clavel et al., 2008) relying on theory indicating that large pitch excurses (large leaps from low pitch to high pitch) often accompany expressions of happiness, while reduced pitch excurses are often found in depressed individuals (Scherer et al., 2001). Regarding textual representations, the word/n-gram frequencies in the document are easily tuned using previous linguistic knowledge on emotional states through existing resources (Jin et al., 2009) or existing rules already identified in systems presented in Section 3.1. For example, in Raphalen et al. (2022), hedging features were designed from linguistic theories and prove their efficiency for the detection of different types of hedges in conversations. Here conversational knowledge is implicated at the front end, when the feature scheme is chosen.

On the other hand, representations obtained by previously training a model on unlabeled text mostly aim to give the most theory-independent representation of features, while depending in a general way on the notion that units of a phrase can be predicted from their neighbors. These powerful representations are learned from a huge amount of data in what is usually called self-supervised learning: by definition, a self-supervised process will not take into account the fact that emotions are what is being predicted. For almost the last decade, the trend has been to pre-train these representations with models such as Word2Vec (Mikolov et al., 2013) or BERT (Devlin et al., 2018), to be re-used into supervised tasks, where labeled data is scarce. This is called fine-tuning: features obtained from self-supervision are adapted to the task at hand. This second phase is where social science-derived labels (such as specific emotions) may be integrated (Zhou J. et al., 2020) (end-to-end recognition). This new paradigm can be applied to both modular and end-to-end dialogue systems: indeed, we can pre-train open-domain generative models then fine-tune them: 1) to predict the internal information necessary to determine a dialogue policy such as emotions for empathy strategies (Siriwardhana et al., 2020), or, 2) to generate or to rank utterances conditionally to specific conversation strategy (Hu et al., 2020; Zhong et al., 2020; Liu et al., 2021).

### 3.3 How can we integrate the two perspectives?

Famous statistician George Box said, 40 years ago, “All models are wrong but some are useful” (Box, 1979). More recently, as vice-president of research at Google, Peter Norvig has argued that “if the model is going to be wrong anyway, why not see if you can get the computer to quickly learn a model from the data, rather than have a human laboriously derive a model from a lot of thought”^5^. Norvig’s comment explains the position of the companies and think tanks that build large end-to-end language models based on massive amounts of data. Neural models are indeed now very powerful and useful to model some aspects of dialogues. However, since the release of GPT-3, it has become increasingly clear that if a system is to sustain a conversation with human users, simply relying on data to generate responses is not sufficient.

We argue, along with others, that because conversational interactions among humans always involve a social dimension as well as task, systems must bring together neural models with what the social sciences can teach us about conversation. Such hybrid systems can best leverage the full potential of neural network research. When social-science derived supervision intervenes in neural models, we should develop methods able to deal with smaller labeled datasets, because annotating interactions in social labels is more tricky and costly than annotating cats in images. We saw that when working on small corpora, studies favor machine learning models with social-science derived features. Regarding neural models, one promising approach, beginning to be found in the literature, is to integrate social science-based rules as features in the encoding stage. Similarly pre-trained representations using a knowledge base from the social science literature can be integrated into graph neural models (Li et al., 2021). In addition, these hybrid approaches have the benefit of allowing greater explainability of the output. However, research on neural models and NLP offers methods such as meta-learning (Deng et al., 2020), few-shot learning (Fei-Fei et al., 2006) and multi-task learning (Ruder, 2017) in order to foster the tractability of the models. The application of such methods for the analysis of conversations is emerging and not trivial. As an example, in Guibon et al. (2021), we showed that a meta-learning approach using prototypical networks improved results for analyzing emotion in a small dataset of conversations. But the obtained results were still lower than when using a model trained on a bigger version of the same dataset. We should keep on leveraging such research in order to take advantage of all the already existing corpora where social phenomena are annotated. We also believe that a better understanding of the relationship between the different social phenomena illustrated in the datasets will improve knowledge transfer between neural models.

The recent trend of using human-in-the-loop feedback in order to update the model is another step towards model control by human knowledge. This framework seems particularly relevant for dialogue models. Human feedback can take numerical form (rewarding a right answer from a task-oriented model) or textual form (Li et al., 2017), and the model can be updated online with reinforcement learning, or offline, for example with adversarial training (Wallace et al., 2019). Besides simple rewards based on user satisfaction (Park et al., 2020), the idea of correcting the behavior of a model by asking a human to rank what has been generated is emerging, with the goal of aligning the model to human intent (Ouyang et al., 2022). However, this exclusively data-driven solution relies on the intervention of many humans, which may be of varying quality. So now we have to work on how to deal with human subjectivity.

In addition, as Microsoft discovered with Tay and XiaoIce (Zhou L. et al., 2020), garbage in equals garbage out. That is, learning in real-time means that, for example, if users of the system spew racist comments, the system will grow to become racist too. Studies are recently carried out in order to tackle this lack of safety in the generated answers by fine-tuning end-to-end neural models on annotated data and by enabling the models to consult external knowledge sources (Thoppilan et al., 2022).

## 4 Challenge 3: Beyond inter-personal, to dyadic

Our final challenge revisits the nature of social functionality in socio-conversational systems. We present here studies that look at both participants as separate individuals trading meanings (interpersonal), and discuss the social science literature that advocates instead considering a conversation as a dyadic process of two interlocutors working together. We then examine avenues for the integration of dyadic processes into interpersonal models.

### 4.1 Inter-personal models

Significant research, called here inter-personal, has looked at the impact of one turn of talk on the next, treating the verbal, paraverbal and visual aspects of one speaker’s turn as context for the next speaker’s utterance. In conversational analysis, and subsequent dialogue system research, this might be referred to as “request/response pairs” or adjacency pairs, although other phenomena are also included (Schegloff, 2007). Classical task-oriented dialogue systems generate utterances as a response to a query (Hovy et al., 2000). Some social-oriented dialogue systems integrate interpersonal aspects across modalities. They look at how to generate a smile in the listener in response to a joke from the speaker (Niewiadomski et al., 2010), or a backchannel during a pause in talk by the interlocutor (Poppe et al., 2010).

In both modular or end-to-end approaches, some of these systems only look at a single prior turn by the user (for example, in some question-answering systems), and some rely on some kind of longer history (in non end-to-end systems, Partially Observable Markov Decision Processes (POMDP) take this approach). Regarding understanding (one of the modules of modular approaches), current neural models usually include neighboring utterances to make their predictions. Most of these models make no distinction as to whether the neighboring utterances were generated by the agent itself or by the human interlocutor. For example, Hazarika et al. (2018) structure a hierarchy of turns, incorporating the particular structure of dialogue, with dependencies mirroring interactions between speakers but also among a speaker’s own utterances. Capturing this kind of contextual dependency between utterances is done through different kinds of neural architectures, such as recurrent neural networks (Poria et al., 2019). Neural architectures also allow information from different modalities to be represented in the same space: a wide array of work has investigated how best to fuse these modalities for dialogue understanding tasks (Zadeh et al., 2017). However, modalities are heterogeneous, often unaligned, and neural networks have difficulty modeling long-range dependencies between them. Modern architectures, such as the attention mechanism, are addressing the issue (Ghosal et al., 2018), with research also investigating how to deal with missing modalities at prediction time (Tang et al., 2021).

Regarding generation, somewhat more sophisticated systems maintain memory of the user’s goal, and attempt to generate responses over the course of several turns to satisfy the user’s goals (Young et al., 2010). More recently, however, the approach to generation in context has changed quite radically. For example, using a GPT-2 model, Cao et al. (2020) study how to efficiently fuse different sources of information with various attention mechanisms in order to generate responses that are coherent not just with the previous turn, but with the history of the conversation, and even the persona of the agent.

### 4.2 Dyadic models

Modeling inter-personal dynamics gives a more natural feel to conversation between humans and agents, in both task-oriented, social-oriented and both task+social systems. However, such interpersonal models take the term “social” rather lightly, as in their context it means phenomena that are somewhat outside of task per se such as emotion, personality, and stance. In contrast to the approach taken by conversational analysis and psycholinguistics, to be truly social, conversation is co-constructed by both interactants together. In this perspective, conversation is itself a collaboration. Phenomena that illustrate this perspective include conversational grounding (Clark, 1996) where speakers attend to the listener’s uptake of information, and change the content of their utterances in real time to rectify and clarify what is said. Other phenomena include rapport where, it has been argued, interlocutors observe the verbal, nonverbal and paraverbal behaviors of their interlocutors as they assess the strength of the social bond between them, and adjust their utterances to either strengthen, maintain or destroy that bond, depending on their goals (Zhao et al., 2014). The phenomena of conversational entrainment, convergence and alignment demonstrate that interlocutors increasingly synchronize or even mimic one another’s language and nonverbal behaviors. This too requires calculating the behavior of the dyad, rather than the behavior of an individual (Chartrand and Bargh, 1999). We believe that this too is an essential future direction for conversational systems that wish to engage human users (Eskenazi and Zhao, 2020).

In contrast to processes studied in interpersonal models (e.g., sentiment), dyadic processes are not observable at the level of a turn. They are evolving processes that are measured over longer periods of time. Computing models of dyadic processes have been designed to automatically analyze conversations (Langlet and Clavel, 2015; Zhao et al., 2016; Madaio et al., 2017; Kantharaju et al., 2020). What these models have in common is that their unit of analysis is the dyad formed by the two participants (whether they are agents or people or a combination of the two). What these models differ on is the duration of the unit of analysis, which depends on the particular dyadic processes. In Langlet and Clavel (2015), the dyadic process studied is shared likes and dislikes and the unit of analysis is rather short with segments based on two adjacent turns (adjacency pair). In Madaio et al. (2017); Zhao et al. (2016), the dyadic process in question is rapport and the unit of analysis is much longer with 30-s segments containing turns of the two participants. This unit of analysis can be even longer (2-min segments) when studying phenomena such as cohesion (Kantharaju et al., 2020).

The above measures of dyadic processes have only infrequently been integrated into socio-conversational systems until now. However, they do exist, including the implementation of various alignment processes at both the emotional [social/emotional resonance in Gratch et al. (2013)], verbal (Duplessis et al., 2021) and non verbal levels [mimicry in Philippot et al. (1999)]. The objective is to use such processes to improve the user’s perception of the agent’s competence and the user’s performance on tasks, relying on previous studies of the link between alignment and both social competence (Pfeifer et al., 2008) and performance (Sinha and Cassell, 2015). For example, in Verberne et al. (2013), the authors discover links between mimicry and a user’s liking of and trust towards the agent. In Campano et al. (2015), verbal alignment is triggered depending on a real-time measure of user engagement.

Other studies prefer to focus on the implementation of nonverbal or verbal conversational behaviors directly dedicated to strengthen dyadic processes such as social bonds with the users. Such studies identify conversational strategies that can, either *a priori* or based on human-human research, strengthen social bonds and then evaluate the effect the conversation strategies have on the user at the end of the interaction. In Baker et al. (2018); Tolmeijer et al. (2020), reparation strategies are implemented on the agent’s side with the aim of fostering trust (ex. Trust repair such as apology, explanation or denial). Conversation strategies such as self-disclosure and *reciprocity* are implemented in Lee and Choi (2017); Bickmore et al. (2013) to improve user experience and strengthen social bonds for two different applications: movie recommendation in Lee and Choi (2017) and a museum guide agent in Bickmore et al. (2013).

### 4.3 How to merge interpersonal and dyadic models?

An agent’s utterance always comes after a user’s turn and in reaction to what has happened previously, so segmentation into turns of speech is necessary for generation models. Interpersonal models allow a wider context to be taken into account in deciding the content of the agent’s utterance (the context related to the inter-turn dynamics as analysed in the dialogue history). To incorporate dyadic processes in these models, the context used will have to be based on real-time measurement of dyadic processes. First, this implies units of analysis that are not an interlocutor or a message but a dyad or a group. Novel coding schemes and computational models all must be adapted to take into account the dyadic nature of these phenomena [e.g., computational models of rapport level for each 30-s frame in Zhao et al. (2014)]. The various neural architectures developed for interpersonal models and presented in Section 4.1 can be leveraged with a different kind of supervision than currently (e.g., using the speaker turn as a unit and basic emotion categories as a label) and by working on the last layers of the neural architectures in order to integrate the slowly evolving nature of dyadic processes. It is this evolution that determines the choice of conversational strategies.

Existing socio-conversational systems that use dialogue strategies targeting dyadic processes do not for the most part take advantage of these measures. They more often focus on generating behaviors that are likely to elicit rapport *a priori* (e.g., teasing) but do not adapt to the dyad as it emerges in the interaction. The consequence is that the timing may not be right (the agent teases at an inappropriate moment). Some research based on modular architectures does however begin to suggest ways in which the agent’s actions can be decided based on these measures [alignment measures in Duplessis et al. (2021) and rapport measure in Zhao et al. (2018)]. Perhaps the most promising approach is to use reinforcement learning with a reward for fostering dyadic processes. This is in line with what has been proposed recently by Pecune and Marsella (2020).

Meanwhile, research on interpersonal models also proposes interesting GPT-like neural architectures that have the potential to integrate dyadic processes. It remains to be seen how this integration can be achieved and in particular how multimodal information related to dyadic processes can be integrated in end-to-end neural architectures dedicated to generation. An interesting approach would be to introduce supervision into the generation process of end-to-end models of that kind, in order to train the system to generate the answers that foster dyadic processes across modalities.

When the aim is sustaining a long-term relationship with the user, the ability of the system to analyze and calibrate the user-agent social relationship to a long history of previous interactions gains in importance. So far, the focus has been on how to build an incremental model of the user across more than one interaction (Bickmore et al., 2010). More recent work has focused on the strength of the user-agent social relationship over a prolonged usage of the system through complex modular approaches, such as proposed in De Visser et al. (2020). It is not yet clear how this integration of the history of the user-agent relationship translates into end-to-end generation approaches for socio-conversational systems. However, the memory networks and knowledge base memory managers in neural architectures that have been recently proposed in order to keep track of long dialogue could be a good way forward (Wang et al., 2020).

## 5 Discussion

The three above-mentioned challenges contribute to the development of socio-conversational systems that provide fluid, natural, and efficient interactions. What emerges from these challenges is that we need research that does not remain confined to a single type of method, leveraging different research fields such as natural language processing, affective/social computing and social science.

First, there is a need to better mix task and social. Whereas task-oriented systems tend to ignore the social aspects, social-oriented systems are more frequently linked to a task. But the tasks that are targeted by social-oriented systems are often restricted to “socially-oriented task”, such as museum guide agents or tutoring agents for education applications. We argued here that more conversational systems need to integrate the social aspects of the interaction: even for train reservation systems, we need to ensure that the user is not left frustrated by the interaction. Social-oriented and task-oriented systems rely on similar modular architectures. However, when it comes to end-to-end generation models, if they are performing well for question-answering systems, they are more complicated to deploy for social-oriented systems, because we partially lose control over the response content.

Second, other ways of integrating information from the social sciences could be explored by intervening within the neural architecture itself. Until now, information from the social sciences has been integrated into the neural architectures used for socio-conversational systems either in the form of supervision or in the form of multimodal cues to be integrated into the representations at the input of the architectures. However, supervised models are greedy in labeled corpora that are rather scarce when it comes to labels of social phenomena. Using hybrid approaches would allow us to take advantage of the performance gain offered by neural models while keeping control.

Third, we have seen that interpersonal models offer interesting neural architectures that take into account interpersonal dynamics. We have also examined the role of dyadic processes in an interaction and what socio-conversational systems could gain by integrating them. However, there is still research to be done in order to integrate these dyadic processes within interpersonal models.

Tackling these three challenges should be done jointly because they are intertwined. Interleaving task and social functionalities (Challenge 1) requires finding new ways of integrating social science knowledge in neural architectures (through supervision is not the single option) (Challenge 2). The social functionality in Challenge 1 should also be revisited: instead of considering the generation of basic emotions or specific stances, we need to target the generation of verbal and non verbal behaviors that are at the heart of dyadic processes described in Challenge 3. To do so, we need to leverage architectures behind interpersonal models for the integration of dyadic processes (Challenge 3), which requires to find the right balance between fully end-to-end generation models and rule-based models for a smarter and multi-level coupling of analysis and generation (Challenge 2) and between the relative importance given to the task and the social aspect (Challenge 1).

## Footnotes

1 In this Indian story, a group of blind men imagine what an elephant looks like, but each is touching a different part of the elephant’s body leading to very different descriptions.

2 Other researchers have recently launched similar calls to capture the dyadic and co-construction feature of conversation Kopp and Krämer (2021); Eskenazi and Zhao (2020); Benotti and Blackburn (2021).

3 Note that in the case of spoken interactions, an initial step of automatic transcription of user utterances is required.

4
https://www.kuki.ai/research

5 From https://norvig.com/fact-check.html
